# Cyclic Thermal Shock Damage Behavior in CVI SiC/SiC High-Pressure Turbine Twin Guide Vanes

**DOI:** 10.3390/ma14206104

**Published:** 2021-10-15

**Authors:** Xiaochong Liu, Xiaojun Guo, Youliang Xu, Longbiao Li, Wang Zhu, Yuqi Zeng, Jian Li, Xiao Luo, Xiaoan Hu

**Affiliations:** 1 National Key Lab of Thermostructure Composite Materials, Northwestern Polytechnical University, Xi’an 710072, China; liuchong@nwpu.edu.cn; 2 AECC Hunan Aviation Powerplant Research Institute, Zhuzhou 412000, China; gxj608@163.com (X.G.); nikki27@163.com (Y.Z.); lijiannpu@163.com (J.L.); luoxiaodut@163.com (X.L.); 3 College of Civil Aviation, Nanjing University of Aeronautics and Astronautics, No. 29 Jiangjun Avenue, Nanjing 211106, China; 4 School of Materials Science and Engineering, Xiangtan University, Xiangtan 411105, China; wzhu@xtu.edu.cn; 5 School of Aircraft Engineering, Nanchang Hangkong University, Nanchang 330063, China; hu_xiao_an@163.com

**Keywords:** ceramic-matrix composites (CMCs), SiC/SiC, twin guide vane, thermal shock, damage

## Abstract

In this paper, the SiC/SiC high-pressure turbine twin guide vanes were fabricated using the chemical vapor infiltration (CVI) method. Cyclic thermal shock tests at different target temperatures (i.e., 1400, 1450, and 1480 °C) in a gas environment were conducted to investigate the damage mechanisms and failure modes. During the thermal shock test, large spalling areas appeared on the leading edge and back region. After 400 thermal shock cycles, the spalling area of the coating at the basin and back region of the guide vane was more than 30%, and the whole guide vane turned gray, due to the formation of SiO_2_. When the thermal shock temperature increased from 1400 to 1450 and 1480 °C, the spalling area of the basin and the back region of the guide vane did not increase significantly, but the delamination occurred at the tenon, upper surface of the guide vane near the trailing edge of the guide vane. Through the X-ray Computed Tomography (XCT) analysis for the guide vanes before and after thermal shock, there was no obvious damage inside of guide vanes. The oxidation of SiC coating and the formation of SiO_2_ protects the internal fibers from oxidation and damage. Further investigation on the effect of thermal shock on the mechanical properties of SiC/SiC composites should be conducted in the future.

## 1. Introduction

High turbine inlet temperature (TIT) is a major feature of advanced gas turbine aeroengines. Now, the TIT has increased to 1400–1480 °C [1]. The performance of the superalloy turbine rotor is close to the material limit, so it is difficult to meet the needs of future turbine aeroengines to significantly reduce weight and improve temperature. As the key technology of aerospace development in the future, ceramic-matrix composites (CMCs) will become the in-demand material for aeroengine manufacturing. Different from the existing superalloy material system, CMCs can work at 1650 °C, which can not only reduce the aeroengine weight, reduce the fuel consumption, increase the temperature in front of the turbine and prolong the service life, but it is also an ideal aeroengine hot-section component. At present, CMCs are mainly used in static hot-section components of aeroengines, e.g., the nozzle, low-pressure turbine guide vane, and nozzle regulator. In the future, the main application targets are the high-pressure compressor, high-pressure turbine blade, and guide vane, etc. After 40 years of development, CMCs have already been successfully applied to many types of aeroengines and realized in the engineering production. CMCs have excellent comprehensive performance and a wide application prospect, but it will face the problem of failure in service. At present, CMC performance research mainly focuses on mechanical properties, high temperature oxidation resistance and thermal shock resistance. The research on the performance evaluation system and life evaluation model is still in the basic research stage, especially for the hot-section components under high temperature oxidation and corrosion service environments. The application scope, reliability, and service life still need to be improved [2,3,4].

CMCs are anisotropic materials, which are mainly composed of a fiber-reinforced phase and matrix continuous phase. The two-phase materials will produce different deformation at high temperatures, especially when the temperature field is non-uniform, the mismatch of material deformation will seriously affect the structural stability of components [5]. The service characteristics of high and low temperature alternation of aero engine are extracted as thermal shock performance assessment [6,7,8]. Thermal shock resistance is the key problem to be solved when CMCs are applied to aeroengines [9]. Kastritseas et al. [10] investigated the onset of multiple matrix cracking in unidirectional fiber-reinforced CMCs subjected to thermal shock and established the relationship between the critical quenching temperature differential, processing temperature of the composite and the material properties. Wang et al. [11] investigated the thermal shock behavior of three types of two-dimensional CMCs (i.e., Nicalon^TM^ SiC fiber-reinforced polymer impregnation and pyrolysis (PIP) SiC matrix, Nextel^TM^ 312 fiber-reinforced chemical vapor infiltrated (CVI) SiC matrix, and Nicalon^TM^ SiC fiber-reinforced CVI SiC matrix) using the water-quench technique. The damage of matrix cracking and delamination induced by thermal shock was determined using the flexure testing and dynamic resonance measurement. Udayakumar et al. [12] investigated the effect of thermal cycling of 2D plain-woven SiC/SiC composites on the mechanical properties, e.g., the proportional limit stress, percentage elongation, fracture toughness, and interfacial bonding strength. Zhang et al. [13] investigated the effect of thermal cycles between 900 and 300 °C in air atmosphere on the tensile properties of 2.5D woven C/SiC composite. The composite can retain the tensile strength within 40 thermal cycles. On the fractured surface, extensive fiber’s pullout appeared, indicating the weakening of the bonding strength at the fiber/matrix interface subjected to thermal cycles. Yang and Liu [14] performed cyclic thermal shock test on the oxide/oxide CMCs. The thermal aged CMCs under cyclic thermal shocks showed a rapid decrease in the elastic modulus compared to the original CMCs. Absi and Glandus [15] developed an improved method for the severe thermal shock testing method of ceramcis by water quenching. Jiao et al. [16] performed water quenching thermal shock tests on SiC/SiC guide vanes with different laminates and evaluated the defects in the vanes and thermal shock performance. For the unidirectional laminate SiC/SiC guide vane, the thermal shock resistance is the worst, and the fracture of the guide vane occurs after 10 thermal shocks; however, for the orthogonal laminates, the guide vane possesses better thermal shock resistance with more than 30 thermal shocks. Boccaccini [17] predicted the critical temperature difference for the thermal shock resistance oof different fiber-reinforced CMCs. Li [18,19] analyzed the thermal fatigue damage evolution in fiber-reinforced CMCs, and predicted the tensile stress-strain curves of different CMCs after thermal fatigue loading. Yang et al. [20] developed a damage constitutive model for thermal shocked Oxide/Oxide CMCs. However, in the research mentioned above, the cyclic thermal shock damage behavior in fiber-reinforced CMC components has not been investigated.

The objective of this paper is to fabricate the SiC/SiC high-pressure turbine twin guide vane using the CVI method and perform the cyclic thermal shock tests at different target temperatures (i.e., 1400, 1450, and 1480 °C) in a gas environment. The surface temperature field of the SiC/SiC twin guide vanes is analyzed and the damage characteristics of the composite guide vane after experiencing different thermal shocks are analyzed.

## 2. Fabrication of SiC/SiC Twin Guide Vanes and Cyclic Thermal Shock Experimental Procedure

### 2.1. Fabrication of SiC/SiC Twin Guide Vanes

**Figure 1:**
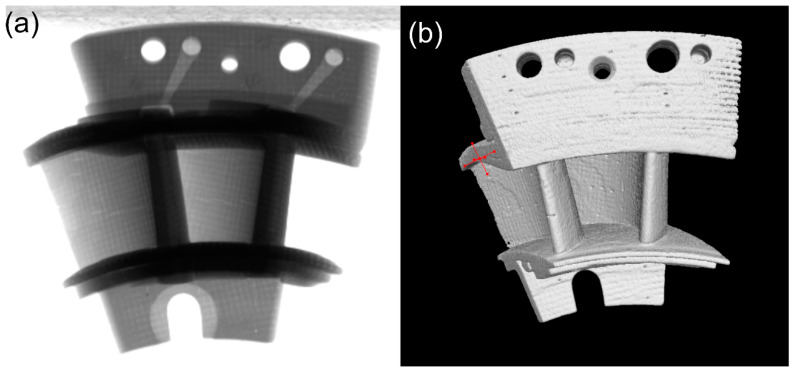
The photograph of SiC/SiC twin guide vanes observed under XCT (**a**) Front view; and (**b**) Back view.

The SiC/SiC high-pressure turbine twin guide vanes were fabricated using the chemical vapor infiltration (CVI) method. The twin guide vanes were formed by 2D lamination. The process of stacking and layering plain-woven cloth and Z-direction puncture and suture was adopted. The fiber’s volume fraction is 40–45%. The SiC fiber’s diameter was 14−16 μm and the density is approximately 2.75 g/cm^3^. To protect the SiC fiber and adjust the thermal misfit between the fiber and the matrix, the PyC interphase was deposited on the surface of the fiber using the chemical vapor deposition (CVD) method. The CVD temperature was approximately 900–1100 °C using the Ar as the protective gas. The deposition time is approximately 20–50 h and the thickness of the interphase is approximately 300–500 μm. After deposition of the interface, the SiC matrix was deposited on the fiber’s preform using the CVI method at approximately 1100–1400 °C. The deposition furnace was vacuumized to 20–50 kPa, 60–100 L/min H_2_ gas was used as carrier gas, the precursor gas flow rate of SiC matrix was 100–500 L/min, and the single deposition time was 100–150 h. When the density of the material was more than or equal to 2.5 g/cm^3^, the densification process was completed. The surface of the SiC/SiC guide vane was then deposited the SiC coating using the CVD method at approximately 1000 °C. Figure 1 shows the photograph of the SiC/SiC twin guide vanes observed under X-ray Computed Tomography (XCT).

### 2.2. Cyclic Thermal Shock Experimental Procedures

**Figure 2:**
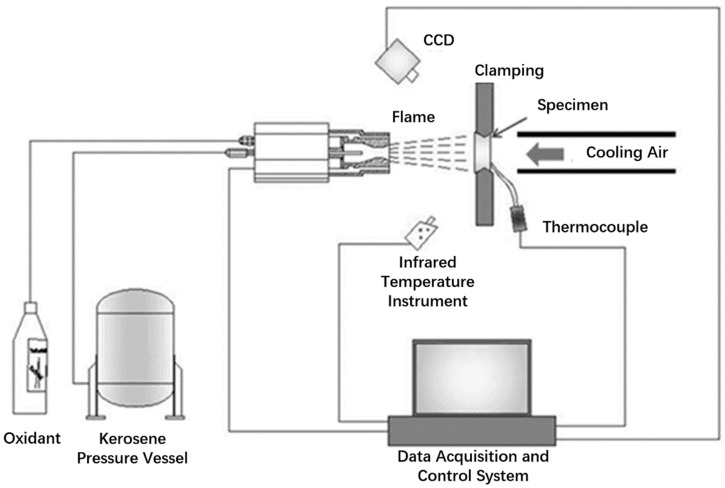
Schematic of function principle for the service environment simulation device.

**Figure 3:**
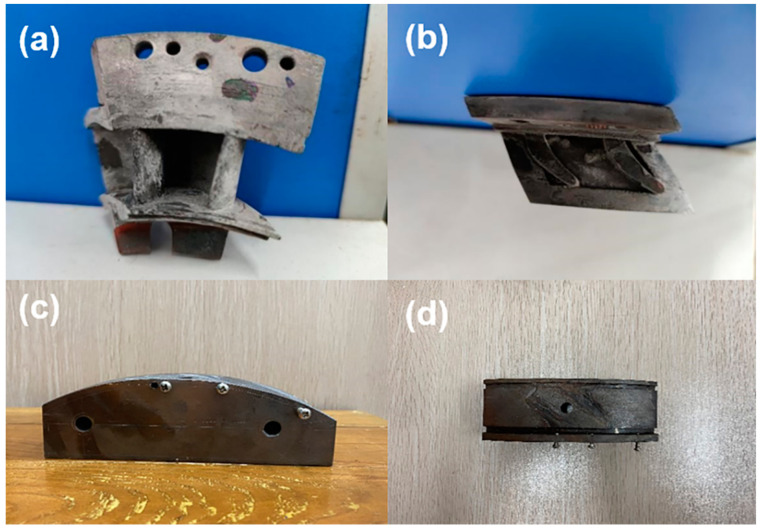
(**a**) Front view of the guide vane; (**b**) Top view of the guide vane; (**c**) Front view of the fixture; and (**d**) Top view of the fixture.

The thermal shock experiments of the CVI SiC/SiC high-pressure turbine guide vane were carried out on the third-generation turbine blade service environment simulation device, developed by Xiangtan University, which is designed to simulate the high temperature, erosion, and corrosion service environment of the turbine blade. It can simulate single or any two or three kinds of service environment, such as alternating temperature cycle, impact of hard particles, and erosion of corrosive gas, etc. Figure 2 shows the schematic of the function principle for the service environment simulator device. The mixture of kerosene and oxygen in the flame gun was ignited using the high-voltage electric spark. Alumina particles were selected as erosion particles, the erosion particle rate was adjustable from 2 to 250 g/min, and the diameter of erosion particles was 10−300 μm. The particle or flame flow rate is adjustable from 20 to 600 m/s, and the maximum experimental temperature of the sample can reach more than 1700 °C. The SiC/SiC twin guide vanes were placed in the high temperature zone and kept for a specified time, and then transferred to the low temperature zone for cooling. In the simulation environment of gas thermal shock, the number of cycles of coating failure on SiC/SiC turbine twin guide vanes’ surface was determined. The installation method of SiC/SiC twin guide vanes for high temperature cyclic thermal shock test is shown in Figure 3. The SiC/SiC twin guide vanes are fixed on the fixture matching the radian of the bottom guide vane. The bolt passes through the guide vane edge and is fixed with the fixture. There is a hollow hole from the bottom to the top of the fixture to guide the cooling air into the vane, and the cooling air flows out from the cooling channel inside the vane to provide the cooling air inside the guide vane.

**Figure 4:**
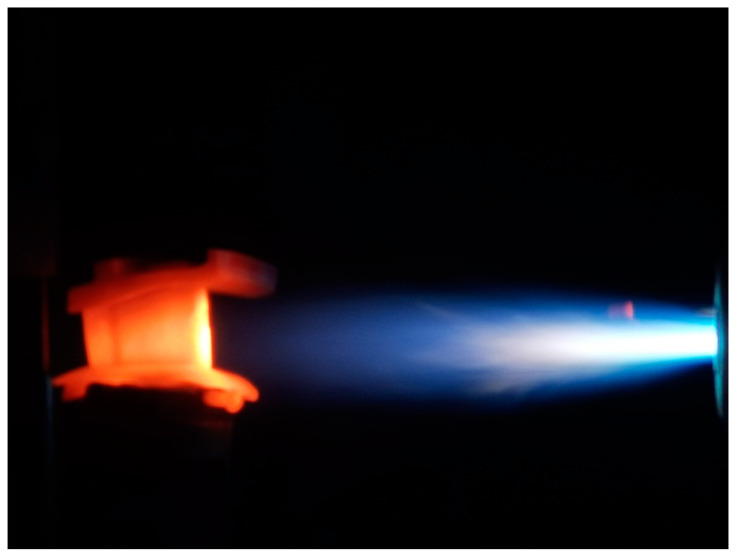
Thermal shock experiment for the SiC/SiC turbine twin guide vanes.

The high temperature gas impacts the twin guide vane in the 90° angle. The infrared thermometer MR1SBSF (Raytak, Santa Cruz, CA, USA) was used to monitor the surface temperature of twin guide vanes and record the real-time temperature at the guide vane’s surface and the flame. The temperature at the guide vane’s leading edge middle section is set to be *T* = 1400, 1450, and 1480 °C with the heating time *t* = 30 s, holding time *t* = 30 s, and the cooling time *t* = 60 s. The inlet pressure of the oxygen in the flame gun was 1.24 MPa, and the flow rate of the oxygen in the flame gun was 180, 200, and 230 L/min for the target temperature of 1400, 1450, and 1480 °C. The inlet pressure of the kerosene in the flame gun was 0.6 MPa, and the flow rate of the kerosene was 5, 6, and 7 L/min the target temperature of 1400, 1450, and 1480 °C. For the testing temperature at *T* = 1480 °C, another thermal shock test with the heating time *t* = 30 s, holding time *t* = 60 s, and the cooling time *t* = 90 s is also conducted. The inlet pressure of cooling channel of the guide vane is *P* = 0.7~0.79 MPa, and the inlet flow rate of cooling channel is *ρ* = 60 L/min or 77 g/min, and the temperature for the cooling air is *T* = 25 °C. Figure 4 shows the figure of the thermal shock test for the turbine guide vane. After cyclic thermal shock tests, the damage regions are observed under scanning electronic microscope (SEM).

## 3. Experimental Results and Discussion

In this section, the surface temperature distribution of the SiC/SiC twin guide vanes under thermal shock test is obtained for different regions, and the damage evolution in the SiC/SiC composite guide vane for different thermal shock tests are analyzed.

### 3.1. Surface Temperature Distribution for the SiC/SiC Twin Guide Vane under Thermal Shock Test

**Figure 5:**
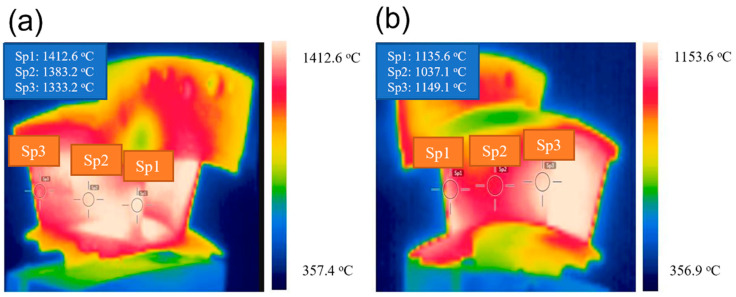
Surface temperature distribution for the twin guide vanes under thermal shock test at the target temperature of *T* = 1400 °C (**a**) Basin area of the left guide vane; and (**b**) Back area of the right guide vane.

Figure 5 shows the infrared thermal imaging of twin guide vanes under thermal shock test at the target temperature *T* = 1400 °C. Due to the shielding of the guide vane, only part of the temperature field of a single guide vane can be monitored and measured (i.e., the basin area in the left guide vane and the back area in the right guide vane). For the left guide vane, the temperature in the middle of the leading edge was the highest, i.e., *T* = 1412 °C. The temperature at the basin area decreased gradually from the leading edge to the trailing edge, and the temperature of the trailing edge was the lowest. For the right guide vane, the temperature of the trailing edge and leading edge was the highest, and the temperature of the middle region was the lowest. The temperature at the basin area was higher than that at the back area of the guide vane.

**Figure 6:**
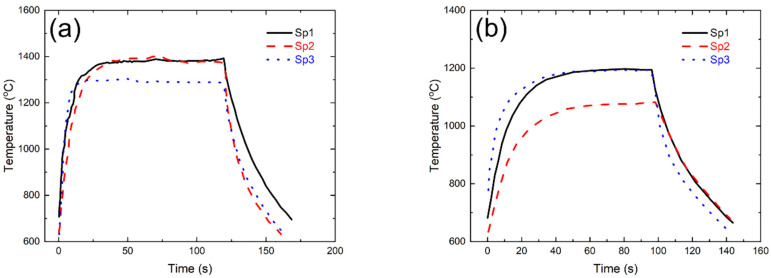
The temperature change with time for different measurement spots during thermal shock test (**a**) Left guide vane; and (**b**) Right guide vane.

Figure 6 shows the extracting infrared temperature data of six measuring points for the left and right guide vanes. It can be found that the temperature at the leading edge of the left guide vane is the highest, and the temperature at the back area of the right guide vane is the lowest, which is consistent with the cloud chart of the temperature distribution of infrared thermal imaging. The measurement positions of the left guide vane can be heated to 1400 °C of the target setting temperature within 30 s and can be well held for 30 s. The temperature value in the holding stage is relatively constant and the fluctuation is small. The heating rate at the back area of the right guide vane is much slower than that at the basin area. After cooling, the temperature value is about 630 °C, and the temperature of other measuring points is lower than the front edge temperature. The maximum temperature at the leading edge of the left guide vane can reach the required 1400 °C, and the temperature fluctuation of each measuring point during the holding stage is less than ±20 °C.

**Figure 7:**
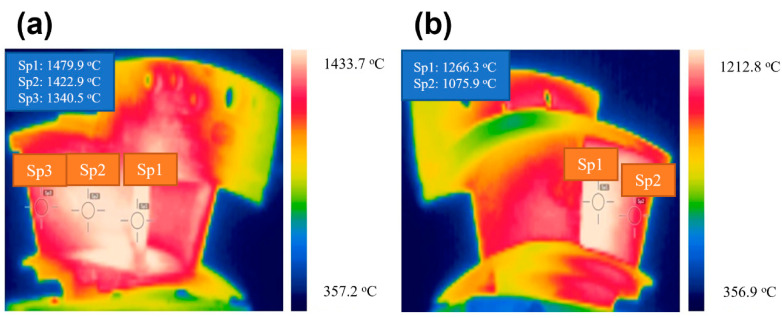
Surface temperature distribution for the twin guide vanes under thermal shock test at the target temperature of *T* = 1480 °C (**a**) Basin area of the left guide vane; and (**b**) Back area of the right guide vane.

Figure 7 shows the infrared thermal imaging of twin guide vanes under thermal shock test at the target temperature *T* = 1480 °C. Due to the shielding of the guide vane, only part of the temperature field of a single guide vane can be monitored and measured (i.e., the basin and back area in the left guide vane). The temperature in the middle of the leading edge was the highest, i.e., *T* = 1479 °C. The temperature at the basin area decreased gradually from the leading edge to the trailing edge, and the temperature of the trailing edge was the lowest. For the back area of the left guide vane, the temperature of the trailing edge was low, and the middle region was high, and the temperature at the basin area was higher than that at the back area of the guide vane.

**Figure 8:**
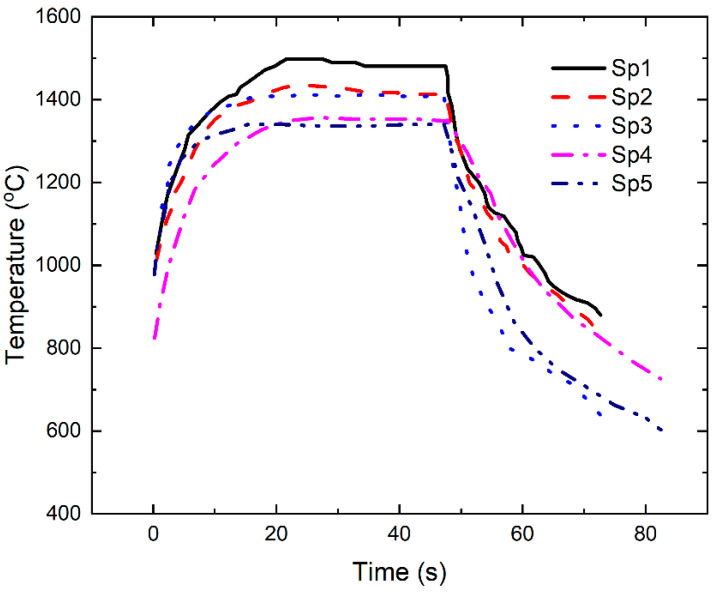
The temperature change with time for five different measurement spots during thermal shock test of left guide vane.

Figure 8 shows the extracting infrared temperature data of five measuring points for the left guide vane. It can be found that the temperature at the leading edge of the left guide vane is the highest, and the temperature at the back area of the left guide vane is the lowest, which is consistent with the cloud chart of the temperature distribution of infrared thermal imaging. The measurement positions of the left guide vane can be heated to 1480 °C of the target setting temperature within 30 s and can be well held for 30 s. The temperature value in the holding stage is relatively constant with small fluctuation. The heating rate at the back area of the left guide vane is much slower than that at the basin area. After cooling, the temperature value is approximately 600 °C, and the temperature of other measuring points is lower than the front edge temperature (i.e., Sp1 in Figure 7a and Figure 8). The temperature fluctuation of each measuring point during the holding stage is less than ±20 °C.

### 3.2. Damage Analysis of SiC/SiC Twin Guide Vanes during Thermal Shock Test

**Figure 9:**
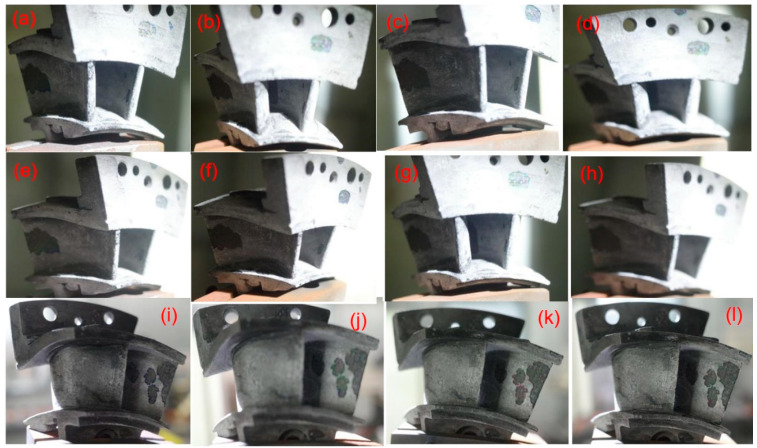
Thermal shock damage of SiC/SiC twin guide vane at target temperature of *T* = 1400 °C for leading edge at (**a**) *N* = 100, (**b**) *N* = 200, (**c**) *N* = 300, and (**d**) *N* = 400, the basin region at (**e**) *N* = 100, (**f**) *N* = 200, (**g**) *N* = 300, and (**h**) *N* = 400, and the back region at (**i**) *N* = 100, (**j**) *N* =200, (**k**) *N* = 300, and (**l**) *N* = 400.

Figure 9 shows the schematic figures for the twin guide vanes at leading edge, basin, and back region after 100, 200, 300, and 400 thermal shock tests. The angle of the thermal shock is 90°, and thus the main thermal shock position of the flame is in the leading edge and the basin region, and the back region of the guide vane is relatively less affected by the high temperature thermal shock.

Before the thermal shock test at *T* = 1400 °C, the surface of guide vane with the SiC seal coating is smooth. With the increase applied cycle number of high temperature thermal shock test, the surface morphology of the back area at the guide vane has no obvious change, however, the coating peeling off in the leading edge at the basin area becomes obvious, which is the peeling off the surface sealing SiC coating of the SiC/SiC guide vane. At *N* = 100 cycles, large spalling areas appeared on the leading edge, the basin, and the back region of the guide vane. After *N* = 400 cycles, the peeling area of the coating at the basin and back area reaches more than 30%, and the guide vane turns gray under the action of high temperature gas. This is due to the formation of SiO_2_ on the surface of SiC/SiC guide vane due to high temperature oxidation under high temperature environment.

**Figure 10:**
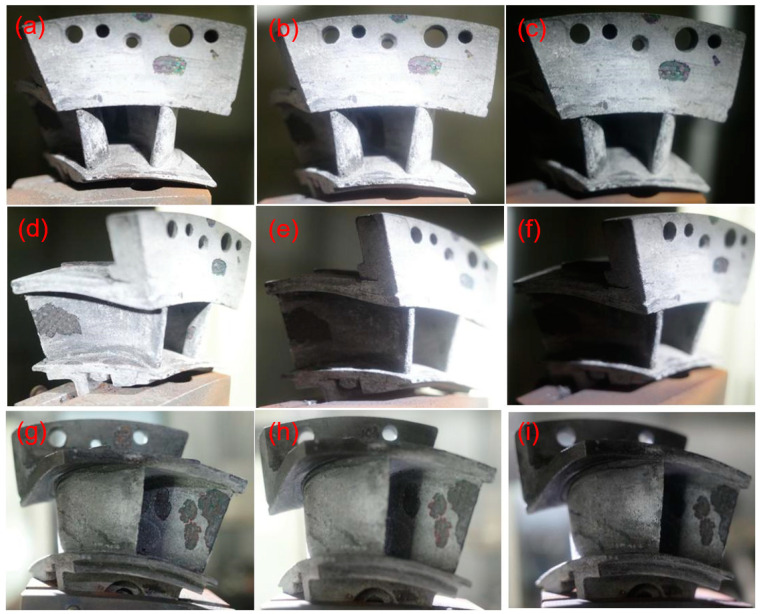
Thermal shock damage of SiC/SiC twin guide vane at target temperature of *T* = 1450 °C for leading edge at (**a**) *N* = 100, (**b**) *N* = 200, and (**c**) *N* = 300, the basin region at (**d**) *N* = 100, (**e**) *N* = 200, and (**f**) *N* = 300, and the back region at (**g**) *N* = 100, (**h**) *N* = 200, and (**i**) *N* = 300.

**Figure 11:**
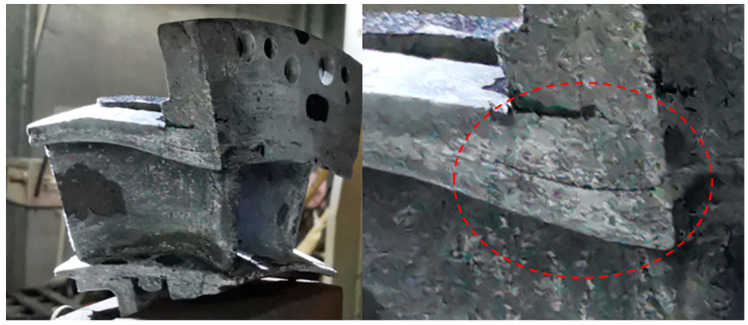
Thermal shock test of SiC/SiC twin guide vane at target temperature of *T* = 1450 °C at *N* = 300.

When the thermal shock testing temperature increased to *T* = 1450 °C, with increasing thermal shock cycle number, the spalling area at the basin and back region of the guide vane was not affected by the increased temperature. Figure 10 shows the figures for the left guide vane at leading edge, basin region, and back region after 100, 200, and 300 thermal shock tests. When the thermal shock cycle number increased to *N* = 300, delamination occurred at the tenon of the left guide vane, as shown in Figure 11. Under thermal shock test, the temperature difference would cause microcracking in the SiC ceramic matrix [17], and the propagation of these microcracks may cause delamination in the components.

**Figure 12:**
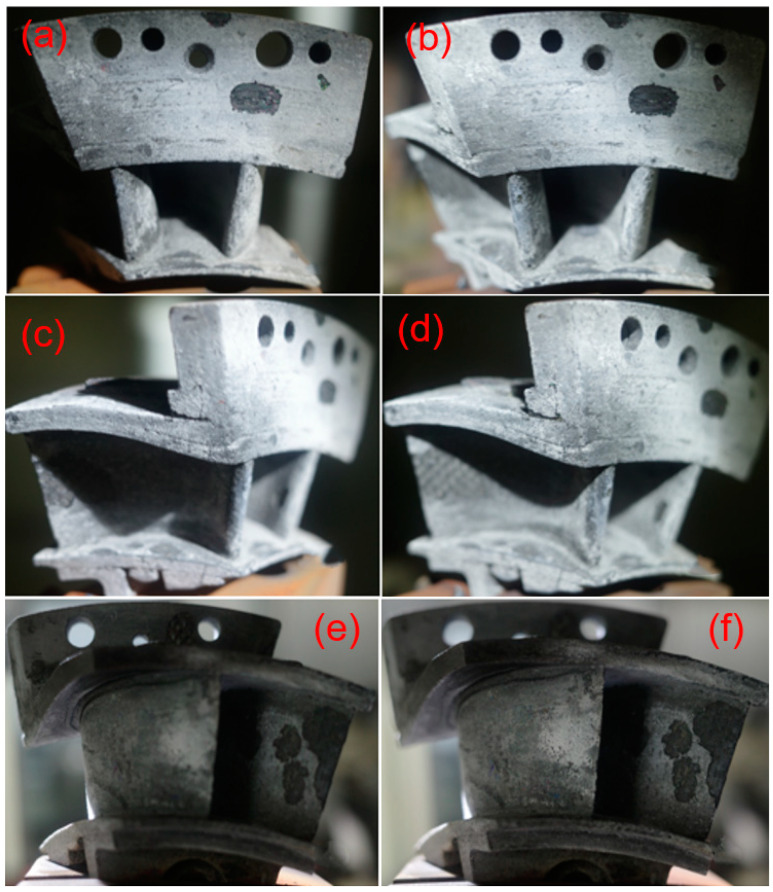
Thermal shock damage of SiC/SiC twin guide vane at target temperature of *T* = 1480 °C with the hold time of *t* = 30 s for leading edge at (**a**) *N* = 100, and (**b**) *N* = 200, the basin region at (**c**) *N* = 100, and (**d**) *N* = 200, and the back region at (**e**) *N* = 100, and (**f**) *N* = 200.

**Figure 13:**
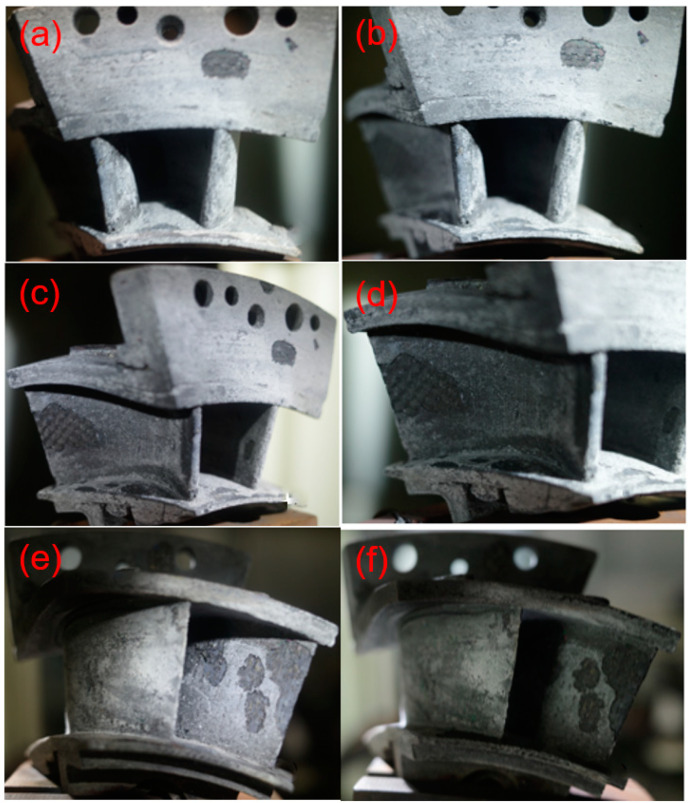
Thermal shock damage of SiC/SiC twin guide vane at target temperature of *T* = 1480 °C with the hold time of *t* = 60 s for leading edge at (**a**) *N* = 100, and (**b**) *N* = 200, the basin region at (**c**) *N* = 100, and (**d**) *N* = 200, and the back region at (**e**) *N* = 100, and (**f**) *N* = 200.

**Figure 14:**
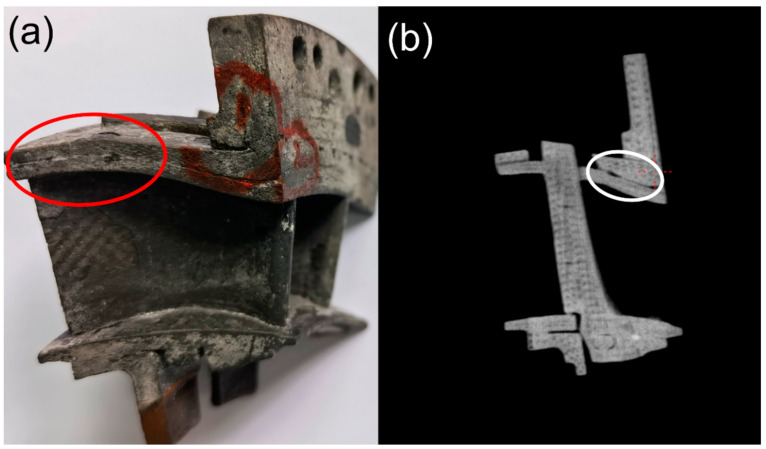
Thermal shock test of SiC/SiC twin guide vane at target temperature of *T* = 1480 °C at *N* = 200 (**a**) New delamination occurred at the trailing edge; and (**b**) New delamination observed under XCT.

Increasing the thermal shock testing temperature to *T* = 1480 °C and increasing the hold time from *t* = 30 to 60 s, the spalling area at the basin and back region did not increase significantly, as shown in Figure 12 and Figure 13. However, at the thermal shock cycle number of *N* = 200, new delamination occurred at the trailing edge of the guide vane, as shown in Figure 14.

## 4. Summary and Conclusions

In this paper, the SiC/SiC high-pressure turbine twin guide vanes were fabricated using the CVI method. Thermal shock tests at the target temperatures 1400, 1450, and 1480 °C in a gas environment were conducted to investigate the thermal shock damage evolution. The surface temperature field of the SiC/SiC twin guide vanes was analyzed and the damage characteristics of the composite guide vane after experiencing different thermal shocks were also discussed.

■ The temperature in the middle of the leading edge of the left guide vane is the highest. The temperature at the basin region decreased from the leading edge to the trailing edge, and the temperature of the trailing edge was the lowest. The temperature at the trailing edge and leading edge of the right guide vane is the highest, and the temperature of the middle region is the lowest. The temperature of the basin region is higher than that of the back region of the guide vane.
■ Before the thermal shock test, the surface of turbine guide vane is smooth. After 100 cycles, large spalling areas appeared on the leading edge and back region, which were the spalling of SiC sealing coating on the surface of SiC/SiC guide vane. After 400 cycles, the spalling area of the coating at the basin and back region of the guide vane is more than 30%, and the whole guide vane becomes gray under the action of high temperature gas, due to the formation of SiO_2_ on the surface of SiC/SiC guide vane under high temperature environment. When the thermal shock test temperature was raised to 1450 °C, the spalling area of the basin and the back region of the guide vane did not increase significantly, but the delamination occurred at the tenon of the guide vane. When the thermal shock test temperature was increased to 1480 °C and the holding time increased from 30 to 60 s, the spalling area of the basin and back region of the guide vane did not increase significantly, but a new delamination phenomenon appeared on the upper surface of the guide vane near the trailing edge.

## Data Availability

The data used to support the findings of this study are available from the paper.
